# Clinical characteristics of tic disorders in children and adolescents with the chief complaint of abnormal blinking

**DOI:** 10.3389/fpsyt.2025.1553358

**Published:** 2025-06-16

**Authors:** Na Tang, Yunjiao Wang, Xiaohan Jiang, Huan Liu, Yan Li, Jia Qu, Shengjin Xiang

**Affiliations:** ^1^ National Clinical Research Center for Ocular Diseases, Eye Hospital, Wenzhou Medical University, Wenzhou, China; ^2^ Key Laboratory of Traditional Chinese Medicine for Myopia Prevention and Control and Chronic Eye Disease Research, Eye Hospital, Wenzhou Medical University, Wenzhou, Zhejiang, China; ^3^ Chengdu University of Traditional Chinese Medicine, Chengdu, Sichuan, China; ^4^ Ineye Hospital of Chengdu University of Traditional Chinese Medicine, Chengdu, Sichuan, China; ^5^ State Key Laboratory of Ophthalmology, Optometry and Vision Science, Eye Hospital, Wenzhou Medical University, Wenzhou, Zhejiang, China; ^6^ National Clinical Research Center for Ocular Diseases, Eye Hospital, Wenzhou Medical University, Wenzhou, Zhejiang, China; ^7^ Research Unit of Myopia Basic Research and Clinical Prevention and Control, Chinese Academy of Medical Sciences (2019RU025), Wenzhou, Zhejiang, China; ^8^ Oujiang Laboratory, Zhejiang Lab for Regenerative Medicine, Vision and Brain Health, Wenzhou, Zhejiang, China

**Keywords:** clinical features, abnormal blinking, tic disorders, allergic conjunctivitis, dry eye disease

## Abstract

**Background:**

Abnormal blinking is a common symptom shared by allergic conjunctivitis (AC), dry eye disease (DED), and tic disorders (TD). This study explored clinical manifestations of TD in patients with the chief complaint of abnormal blinking; its goal was to reduce misdiagnosis and missed diagnosis.

**Methods:**

In total, 1054 patients with the chief complaint of abnormal blinking completed a questionnaire and underwent comprehensive ophthalmic examinations and mental health assessments. Questionnaire data were compiled for patients with a confirmed diagnosis of TD; their clinical characteristics were analyzed.

**Results:**

Of the 1054 patients presenting with abnormal blinking, 453 (42.98%) were diagnosed with a TD. Among these 453 patients, 253 (55.63%) had provisional tic disorder (PTD). 121 (26.71%) patients initially were misdiagnosed (primarily with AC) or experienced a missed diagnosis; Patients with PTD were more likely to seek ophthalmologic care, whereas those with CTD or TS were more likely to visit a paediatrician (P<0.001). The predominant eye tics were excessive and/or frequent blinking; 438 (96.69%) patients exhibited tics other than eye tics. Among the TD patients, 371 (81.90%) reported ocular symptoms, whereas 336 (74.17%) had comorbid eye diseases including AC and DED. PTD patients with AC had higher incidences of allergic rhinitis and asthma compared with patients displaying CTD and TS (P<0.05).

**Conclusions:**

TD are major causes of abnormal blinking, and PTD is the most common subtype. TD patients with abnormal blinking often have comorbid eye diseases, primarily AC and DED.

## Background

Tic disorders (TD) are common neurodevelopmental disorders that typically emerge during childhood and adolescence, with a peak incidence between the ages of 5 and 10. The primary clinical manifestations of TD are sudden, involuntary, repetitive, stereotypical, and nonrhythmic motor and/or vocal tics. These tics often include abnormal blinking, nose wrinkling, facial grimacing, throat clearing, whole-body tics, and accompanying sounds or echoes; all display varying degrees of severity (1, 2). TD encompass a spectrum of conditions, ranging from provisional tic disorder (PTD) to chronic tic disorder (CTD) and Tourette syndrome (TS). These conditions can be distinguished based on the type and duration of tic symptoms (3). The global incidence of TD has been increasing; the estimated prevalences of TS and PTD are 0.77% and 2.99%, respectively. Boys are more frequently affected, and the male-to-female ratio is approximately 3–4:1 (4). In some children, TD symptoms persist into adolescence and adulthood, substantially influencing their quality of life. These individuals often experience behavioural difficulties, such as comorbid obsessive-compulsive disorder (OCD) and attention-deficit/hyperactivity disorder (ADHD). Consequently, TD can cause considerable distress for affected children and their families (1–3).

TD often initially manifest as eye tics (abnormal blinking), which can then gradually progress to facial tics or other types of tics (5). Consequently, patients with TD frequently present to paediatric ophthalmologists with the complaint of “abnormal blinking”, and a positive correlation between eye blinking and TS has been reported (6–10). Comings and Comings (6) reviewed 250 cases of TS and found that the most frequent manifestations were facial tics; excessive blinking was the most common presenting symptom. Jankovic et al. (7) observed excessive blinking in 70% of 156 patients with TS. Tulen et al. (8) found that patients with TS exhibited more frequent blinking compared with healthy controls. In a study of 212 patients with TS, Martino et al. (9) found that 91.5% had an eyelid-related tic, such as blinking, winking, or eye rolling; these tics could sometimes be voluntarily controlled. Nilles et al. (2) examined the clinical characteristics of 203 children with TD, revealing that 56% had eye tics and 48% exhibited eye movement. Collectively, these findings highlight the common nature of abnormal blinking in TS or TD.

Meanwhile, some investigators have also explored the situation where children with abnormal blinking as clinical manifestations were diagnosed with TD. Jung et al. (10) reported that 43(86%) were diagnosed with TD, including 39(78%) cases of PTD. Except for the study, the existing studies have focused on TS (11–13), and all confirmed that TS is very low in abnormal blinking patients, while proposing the concept of “habit tics” (11), “functional blinking” (12), “episodic excessive blinking” (13), respectively. In a retrospective study, only one of 32 children with persistent eye tics was identified TS, but 38% of patients had new motor tics or vocal tics other than eye tics during follow-up, although the authors did not certainly diagnosis of these patients as TD (14). Based on the above results, we believe that the patients with TD is still high in children and adolescence with abnormal blinking. Given the rapid increase in the number of children presenting with abnormal blinking as the chief complaint in paediatric ophthalmology clinics in Asian countries (10), paediatric ophthalmologists should understand the clinical characteristics of TD with abnormal blinking who visit ophthalmology clinics primarily.

Eye diseases may also involve abnormal blinking, the most common of which are allergic conjunctivitis (AC) (15) and dry eye disease (DED) (16). Furthermore, these eye diseases can simultaneously occur with TD, but limited information is available regarding their prevalence in the context of abnormal blinking. Additionally, ophthalmologists may not be sufficiently familiar with the initial symptoms of TD. All of these factors can hinder accurate identification of the cause of abnormal blinking, often leading to delayed diagnosis and misdiagnosis of TD in paediatric ophthalmology clinics (17). Therefore, paediatric ophthalmologists should understand the clinical characteristics of TD with abnormal blinking, particularly in the presence of comorbid eye diseases. This knowledge can facilitate early diagnosis, reducing the likelihood of missed diagnosis or misdiagnosis. This study aimed to summarise the clinical characteristics of TD in patients presenting with the chief complaint of abnormal blinking. It specifically focused on distinguishing between ocular disease-related and TD-associated abnormal blinking, with the goals of improving ophthalmologists’ diagnostic accuracy, reducing rates of misdiagnosis, and improving prevention and management.

## Methods

### Study type

This study is a prospective cross-sectional study.

### Patients and protocol

This prospective study, conducted at the Eye Hospital of Wenzhou Medical University from 1 January 2022 to 31 August 2023, included children and adolescents aged 4 to 18 years who presented with a new complaint of abnormal blinking, and all patients included diagnosed with TD or suspected of TD. Written informed consent was obtained from the parents of all patients. The study adhered to the tenets of the 2013 Declaration of Helsinki and the STROBE statement. The study protocol and informed consent forms were approved by the Medical Ethics Committee of the Eye Hospital, Wenzhou Medical University.

**Figure 1:**
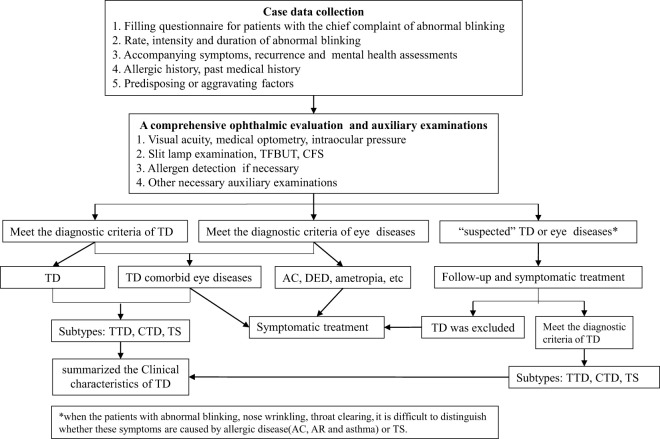
Flow chart of diagnosis and treatment with TD.

Eye blinking is a physiological motor act that can be spontaneous, voluntary, or reflexive. Typically, the blink rate is approximately 10 to 15 times per minute (18). Abnormal blinking refers to a rate and/or intensity of blinking that exceeds the normal range. In present study, abnormal blinking was defined as excessive blinking (refers to an abnormal intensity of blinking, often manifested as eyelid movements such as wrinkling, frowning, eyebrows raising, and eye squeezing), frequent blinking (refers to an abnormal rate or speed of blinking, or the blinking rate exceeds 20 times per minute, or intermittent and continuous blinking), eye rolling, unilateral blinking, or any combination of these symptoms. To accurately assess the clinical characteristics of TD in patients presenting with the chief complaint of abnormal blinking, all patients in our cohort were diagnosed within an ophthalmology clinic. The clinical manifestations included various types of abnormal blinking. All patients had detailed questionnaires filled out by their parents, and data on age, gender, blinking rate, blinking duration, accompanying symptoms, past medical history, recurrence, and attempted treatments. It also included questions about allergies, allergen exposures, and medication history. All patients underwent a comprehensive ophthalmic evaluation, including assessments of visual acuity, medical optometry, intraocular pressure, and slit-lamp examination. All data were recorded using a standardised data collection form. Based on the recorded symptoms and signs, the clinician determined whether the patient had a TD and any accompanying eye diseases. All the clinician were trained in the diagnostic criteria for TD. If a diagnosis could not be established, the patient was scheduled for follow-up visits until a definitive diagnosis could be established. The diagnostic process is illustrated in **Figure 1**.

### Diagnostic criteria

TD diagnoses were made in accordance with the fifth edition of the Diagnostic and Statistical Manual of Mental Disorders (DSM-V) (3). Diagnoses of eye diseases (e.g., AC and DED) were based on relevant guidelines or criteria, as determined by a combination of medical history, physical examination findings, and any necessary ancillary tests. The inclusion criteria were all patients aged 4 to 18 who visited the ophthalmology department with abnormal blinking as the chief complaint. Correspondingly, the exclusion criteria were patients whose age and chief complaint did not meet the inclusion criteria. In addition, if the patient has a history of neurological disorders, such as epilepsy and rheumatic chorea, they will also be excluded.

### Diagnostic principles

To avoid overdiagnosis or misdiagnosis, we established the following diagnostic principles for TD. At the initial visit, TD were diagnosed in patients who presented with tics other than abnormal blinking or who presented with abnormal blinking alone but had a history of TD. These direct diagnoses of TD were made regardless of the presence or absence of accompanying eye diseases. During follow-up, TD were diagnosed in patients who presented with abnormal blinking alone if the blinking persisted or worsened, or if other tics emerged during the follow-up period. For patients with abnormal blinking and eye diseases (e.g., AC or DED), TD were diagnosed if abnormal blinking and/or other tics persisted despite successful treatment of the eye diseases. TD were excluded in patients who presented with abnormal blinking alone and showed a reduction in abnormal blinking during the follow-up period. They also were excluded in patients who presented with abnormal blinking and eye diseases if the abnormal blinking disappeared after successful treatment of the eye diseases. In addition, for those who has eye discomfort but no changes in eye signs, sensory tics should be considered and followed up until TD is diagnosed or ruled out.

Diagnoses of AC were based on a comprehensive assessment of typical symptoms and ocular signs, a history of allergic conjunctivitis or other allergic diseases, and allergen reports. If AC could not be definitively confirmed, the diagnosis was “suspected AC.” During follow-up, if standard anti-allergy treatment led to remission, the patient was diagnosed with AC; otherwise, the diagnosis of AC was excluded.

Considering the difficulty of performing the Schirmer I test in children, diagnoses of DED were based on symptoms, slit-lamp examination findings, tear meniscus height, corneal fluorescein staining, and tear film break-up time (TFBUT). The standard operation for TFBUT detection with corneal fluorescein staining is that the subject blinks three times after dropping the eye drops to ensure thorough mixing of the fluorescein with the tears, and the time between the last blink and the appearance of the first black spot on the cornea is timed with stopwatch, and the average of 3 measurements is taken as the standard. Asia Dry Eye Society recommends a TFBUT <5 seconds as the optimal cutoff for the diagnosis dry eye, patients with TFBUT≥5 seconds cannot be diagnosed with dry eye syndrome, based on this criterion. Patients with abnormal TFBUT but no other symptoms were diagnosed with asymptomatic DED.

### Therapy and follow-up

All patients with eye diseases received appropriate treatment. Patients with PTD alone primarily received mental health education and attended follow-up visits. Patients with CTD or TS were treated with Chinese herbs and/or acupuncture. All undiagnosed patients who with suspected TD, including those with abnormal blinking alone or with eye diseases, underwent treatment and monitoring until a definite diagnosis or exclusion of TD was made during the follow-up period. Similarly, “suspected AC” was diagnosed or excluded based on the results of treatment.

### Statistical analyses

Data were analysed using SPSS 25.0 software. Continuous variables are presented as means ± standard deviations; they were analysed using the independent Student’s t-test and one-way analysis of variance (ANOVA). Categorical variables are presented as frequencies and percentages; they were analysed using the Chi-square test. Multivariable logistic regression analysis was used to explore factors associated with the disease. P-values less than 0.05 were considered statistically significant.

## Results

### Morbidities and demographic characteristics of TD patients

Of the 1054 patients who presented with the chief complaint of abnormal blinking, 508 were diagnosed with a TD or suspected TD. Among these 508 patients, 367 received a clear diagnosis at their initial visit, whereas 141 had a suspected TD. During follow-up, 86 of the 141 patients with suspected TD received a definitive diagnosis; the remaining patients could not be evaluated because their symptoms disappeared or resolved. Finally, 453 patients were diagnosed with TD, constituting 42.98% of the overall cohort; of these, 252 (55.63%) had PTD, 148 (32.67%) had CTD, and 53 (11.70%) had TS. In total, 385 boys (84.99%) and 68 girls (15.01%) were diagnosed TD. The age range was 4 to 18 years (mean, 8.43 ± 2.65 years). Analysis via Chi-squared tests revealed that patients with PTD were significantly younger (P<0.001). The symptom duration ranged from 1 week to 10 years (mean, 19.72 ± 22.28 months). Furthermore, 215 (47.46%) patients were visiting our hospital or another hospital's ophthalmology department for the first time; 238 (52.54%) patients had a history of visiting other hospitals' paediatric departments or had made repeated visits to both ophthalmology and paediatrics departments. Analysis via Chi-squared tests revealed that patients with PTD were more likely to seek ophthalmologic care, whereas patients with CTD or TS were more likely to visit a paediatrician (P<0.001). Additionally, 121 (26.71%) patients initially had a missed diagnosis or were misdiagnosed; PTD was the most common misdiagnosis (P<0.05). Among the patients with missed or misdiagnosed conditions, 86 (71.07%) had allergic diseases (AC or AC and AR), 27 (22.31%) had DED, and eight (6.61%) had other eye diseases.

Among the 453 patients diagnosed with TD, 240 (52.98%) had a past medical history of abnormal blinking. Of these 240 patients, 94 (37.30%) had PTD, 113 (47.08%) had CTD, and 33 (13.75%) had TS. Additionally, 176 (38.85) patients had a past medical history of TD: 60 (34.09%) had PTD, 88 (50.00%) had CTD, and 28 (15.91%) had TS. Specific demographic characteristics are presented in **Table 1** below. Multinomial logistic regression models, using the PTD group as the control, showed that older age and a past medical history of abnormal blinking or TD were significantly associated with higher risks of CTD and TS (P<0.05) (**Table 2**).

**Table 1:** Morbidities and demographic characteristics of TD patients.

Characteristic		TD (n=453)	PTD (252)	CTD (148)	TS (53)	P
Age, years		4-18 8.43 ± 2.65	4-17 7.77 ± 2.39	4-18 9.20 ± 2.92	6-16 9.41 ± 2.20	F=19.16 P<0.001
Gender (%)	Male	385 (84.99%)	211 (83.73%)	126 (85.14%)	48 (90.57%)	χ^2^ = 1.608 P=0.658
	Female	68 (15.01%)	41 (34.21%)	22 (14.86%)	5 (9.43%)	
Course of disease (M)		19.72± 22.28	3.72± 4.03	39.66± 18.88	40.40± 20.68	F=418.96 P<0.001
Department	paediatrics	238 (52.54%)	93 (36.90%)	111 (75.00%)	34 (64.15%)	χ^2^ = 57.511 P<0.001
	ophthalmology	215 (47.46%)	159 (63.10%)	37 (25.00%)	19 (35.85%)	
past medical history of abnormal blinking		240 (52.98%)	94 (37.30%)	113 (76.35%)	33 (62.26%)	χ^2^ = 59.125 P<0.001
past medical history of TD		176 (38.85%)	60 (23.81%)	88 (59.45%)	28 (52.83%)	χ^2^ = 54.816 P<0.001
missed or misdiagnosed diseases		121 (26.71%)	78 (30.95%)	36 (24.32%)	7 (13.21%)	χ^2^ = 7.683 P=0.021*

One-way ANOVA revealed statistically significant differences in age and disease duration among all types of TD (P<0.001). Chi-squared tests showed statistically significant differences in the location (department) of initial diagnosis, past medical history of abnormal blinking, and past medical history of TD (P<0.001).

**Table 2:** Associations between potential influencing factors and risks of TD according to multinomial logistic regression.

Variable	CTD^a^	TS^a^	TS^b^
	*aOR (95%CI)*	*aOR (95%CI)*	*aOR (95%CI)*
Gender			
Male	*Reference*	*Reference*	*Reference*
Female	1.06 (0.55,2.07)	0.58 (0.21,1.63)	0.55 (0.2,1.55)
Age	1.31 (1.19,1.43) ***	1.32 (1.17,1.48) ***	1.01 (0.90,1.13)
past medical history of abnormal blinking	4.12 (2.54,6.67) ***	3.26 (1.72,6.17) ***	0.79 (0.42,1.50)
past medical history of TD	5.99 (3.59,9.99) ***	3.02 (1.57,5.80) **	0.50 (0.25,1.01)

^a^PTD group as control group; ^b^CTD group as control group; aOR, adjusted odds ratio; CI, confidence interval; ***P<0.001; **P<0.01.

### Clinical characteristics

#### Tic symptoms in TD patients

All 453 patients with TD exhibited eye tics (abnormal blinking: excessive blinking, frequent blinking, eye rolling, unilateral blinking). Of these patients, 159 had both frequent and excessive blinking, 145 had frequent blinking, 104 had excessive blinking, 66 had eye rolling, and 75 had unilateral blinking. There were no significant differences in the types of eye tics according to TD subtype. In terms of blink rate, 50 patients exhibited persistent blinking, 262 patients had frequent blinking, and 142 patients displayed occasional blinking. Among all patients, 438 (96.69%) experienced tics other than eye tics. In 98 cases, these other tics preceded the onset of abnormal blinking; in 271 cases, they occurred simultaneously with abnormal blinking; and in 69 cases, they followed the onset of abnormal blinking. The most common tics other than eye tics included nose wrinkling (169, 37.30%), facial grimacing (126, 27.81%), throat clearing (116, 25.61%), mouth tics (115, 25.39%), finger sucking (62, 13.69%), head nodding (61, 13.47%), shrugging (44, 9.71%), neck stretching (43, 9.49%), and other simple or complex tics. Analysis via Chi-squared tests revealed that TS patients were more likely to experience other tics before the onset of eye tics (P<0.05) (**Table 3**); they also more often accompanied by tics other than eye tics (P<0.001) (**Table 4**). Further details are presented in **Tables 3**, **4**.

**Table 3:** Eye tic characteristics in TD patients.

Characteristic		TD (n=453)	PTD (252)	CTD (148)	TS (53)	P
Type of eye tics	frequency blinking	145 (32.01%)	81 (32.14%)	46 (31.8%)	11 (20.75%)	χ^2^ = 4.582 P=0.101
	excessive blinking	104 (22.96%)	55 (21.83%)	39 (26.35%)	18 (33.96%)	
	excessive & frequency blinking	159 (35.09%)	90 (35.71%)	48 (32.43%)	21 (39.62%)	
	eye rolling	66 (14.57%)	39 (15.48%)	21 (14.19%)	6 (11.32%)	
	unilateral blinking	75 (16.56%)	38 (15.08%)	27 (18.24%)	10 (18.87%)	
	other	14 (3.09%)	9 (3.57%)	4 (2.70%)	1 (1.89%)	
	total^#^	563^#^	312^#^	185^#^	67^#^	
Rate of eye tics	persistently	50 (11.03%)	25 (9.92%)	17 (11.49%)	8 (15.09%)	χ^2^ = 3.087 P=0.543
	frequently	262 (57.84%)	152 (60.32%)	79 (53.38%)	31 (58.59%)	
	occasionally	142 (31.35%)	76 (30.16%)	52 (35.14%)	14 (26.42%)	
times of the other tics	before the blinking	98 (21.63%)	55 (21.83%)	24 (16.22%)	21 (39.62%)	χ^2^ = 12.425 P=0.014
	simultaneously with the blinking	271 (59.82%)	155 (61.51%)	94 (63.51%)	23 (43.40%)	
	after the blinking	69 (15.23%)	35 (13.89%)	22 (14.86%)	9 (16.98%)	

Because some patients exhibited two or more types of abnormal blinking, the total number of abnormal blinking instances is not equal to the number of patients.

**Table 4:** Other tic symptoms in TD patients.

Characteristic	TD (n=453)	PTD (252)	CTD (148)	TS (53)	P
nose wrinkling	169 (37.30%)	95 (37.70%)	55 (37.16%)	19 (35.85%)	χ^2^ = 108.647 p<0.001
facial grimacing	126 (27.81%)	73 (28.97%)	38 (25.68%)	15 (28.30%)	
throat clearing	116 (25.61%)	55 (21.83%)	14 (9.46%)	47 (88.68%)	
mouth tic	115 (25.39%)	58 (23.02%)	48 (32.43%)	9 (16.98%)	
fingers sucking	62 (13.69%)	34 (13.49%)	17 (11.49%)	11 (20.75%)	
head nodding	61 (13.47%)	31 (12.30%)	22 (14.86%)	8 (15.09%)	
shrugging	44 (9.71%)	21 (8.33%)	11 (7.43%)	12 (22.64%)	
neck stretching	43 (9.49%)	15 (5.95%)	19 (12.84%)	9 (16.98%)	
leg shaking	28 (6.18%)	13 (5.16%)	8 (5.41%)	7 (13.21%)	
coprolalia	22 (4.86%)	9 (3.57%)	2 (1.35%)	11 (20.75%)	
hand spinning	14 (3.09%)	6 (2.38%)	4 (2.70%)	4 (7.55%)	
shriek	12 (2.65%)	6 (2.38%)	2 (1.35%)	4 (7.55%)	
abdominal tics	11 (2.43%)	5 (1.98%)	1 (0.68%)	5 (9.43%)	
aggressive behaviour	4 (0.88%)	1 (0.40%)	1 (0.68%)	2 (3.77%)	
others	11 (2.43%)	6 (2.38%)	2 (1.35%)	3 (5.66%)	
Total^#^	832^#^	428^#^	244^#^	160^#^	

^#^Because some patients exhibited two or more types of tics, the total number of tic symptoms is not equal to the number of patients.

#### Accompanying ocular symptoms and signs

Among the 453 patients with TD, 371 (81.90%) reported ocular symptoms. The most common symptoms were itching (193, 42.60%), dryness (102, 22.52%), redness (82, 18.10%), fatigue (82, 18.10%), photophobia (58, 12.80%), and eye discharge (42, 9.27%). There were no significant differences in ocular symptoms according to TD subtype. Among these 371 patients, ocular symptoms preceded abnormal blinking in 82 cases, occurred simultaneously with abnormal blinking in 231 cases, and followed the onset of abnormal blinking in 58 cases. Further details are presented in **Table 5**.

**Table 5:** Ocular symptoms in TD patients.

Characteristic		TD (n=453)	PTD (252)	CTD (148)	TS (53)	P
No ocular symptoms		82 (18.10%)	49 (19.44%)	24 (16.22%)	9 (16.98%)	χ^2^ = 13.932 P=0.904
Itching		193 (42.60%)	104 (41.27%)	67 (45.27%)	22 (41.51%)	
dryness		102 (22.52%)	56 (22.22%)	32 (21.62%)	14 (26.42%)	
Redness		82 (18.10%)	46 (18.25%)	25 (16.89%)	11 (20.75%)	
fatigue		82 (18.10%)	41 (16.27%)	27 (18.24%)	14 (26.42%)	
photophobia		58 (12.80%)	29 (11.51%)	19 (12.84%)	10 (18.87%)	
discharge of eye		42 (9.27%)	24 (9.52%)	11 (7.43%)	7 (13.21%)	
sore numb		25 (5.51%)	9 (3.57%)	13 (8.78%)	3 (5.67%)	
pain		24 (5.29%)	10 (3.97%)	9 (6.08%)	5 (9.43%)	
foreign body sensation		22 (4.86%)	14 (5.56%)	6 (4.05%)	2 (3.77%)	
visual blur		18 (3.97%)	11 (4.37%)	4 (2.70%)	3 (5.66%)	
others		4 (0.88%)	2 (0.79%)	2 (1.35%)	0 (0)	
times of the ocular symptoms	before the blinking	82 (18.10%)	46 (18.25%)	27 (18.24%)	9 (16.98%)	χ^2^ = 2.311 P=0.679
	simultaneously with the blinking	231 (50.99%)	132 (52.38%)	71 (47.97%)	28 (52.83%)	
	after the blinking	58 (12.80%)	27 (10.71%)	22 (14.86%)	9 (16.98%)	

#### Accompanying eye diseases

Excluding ametropia (236 cases, 52.10%), 317 TD patients (69.98%) had comorbid eye diseases; of these patients, 180 had PTD, 120 had CTD, and 36 had TS. Overall, ametropia, AC, and DED were the primary eye diseases accompanying TD. Specifically, 164 (36.20%) patients had AC, 92 (20.31%) patients had DED, and 52 (11.48%) patients had both. Analysis via Chi-squared tests revealed significant differences in AC and DED prevalence according to TD subtype. TS patients were more likely to have both AC and DED but less likely to have DED alone (P*<*0.01) (**Table 6**). In contrast, patients with PTD and CTD were more likely to have DED alone. Further details are presented in **Table 6**.

**Table 6:** Accompanying eye diseases in TD patients.

Characteristic		TD (n=453)	PTD (252)	CTD (148)	TS (53)	P
Accompanying eye diseases	Ametropia	236 (52.10%)	150 (59.52%)	62 (41.89%)	24 (45.28%)	
	AC	164 (36.20%)	91 (36.11%)	58 (31.19%)	15 (28.30%)	χ^2^ = 17.505 P=0.002
	DED	92 (20.31%)	50 (19.84%)	38 (25.68%)	5 (9.43%)	
	AC&DED	52 (11.48%)	22 (8.73%)	16 (10.81%)	14 (26.42%)	
	asthenopia	14 (3.09%)	9 (3.57%)	3 (2.03%)	2 (3.77%)	
	trichiasis	20 (4.42%)	11 (4.37%)	7 (4.73%)	2 (3.77%)	
	others	11 (2.43%)	10 (3.97%)	1 (0.68%)	0 (0)	
	total^#^	405^#3^	209^#^	133^#^	46^#^	

^#^Because some patients had two or more comorbid diseases, the total number of comorbid eye diseases is not equal to the number of patients.

### Clinical characteristics of AC and DED in TD patients

Among 216 TD patients with AC (including 52 patients with both AC and DED), 83 exhibited frequent blinking, 71 exhibited excessive blinking, 95 exhibited both frequent and excessive blinking, and 47 exhibited eye rolling. Other allergic diseases were common in this group: 120 patients had allergic rhinitis (AR), and 84 patients had allergic asthma. Analysis via Chi-squared tests showed that PTD patients were more likely to have other allergic diseases (P*<*0.001). Allergen testing was performed in 94 patients, and 58 (59.18%) had identifiable allergens. These mainly included dust mites (37 cases), followed by milk (11 cases). Among 145 TD patients with DED (including 52 patients with both AC and DED), 51 exhibited frequent blinking, 32 exhibited excessive blinking, and 47 exhibited both frequent and excessive blinking. Furthermore, 109 of these 145 patients reported various ocular symptoms, such as dryness, fatigue, and itching. The remaining 36 patients had only a shortened TFBUT without any ocular discomfort. Further details are presented in **Tables 7**, **8**.

**Table 7:** Clinical characteristics of AC in TD patients.

Characteristic		TD (n=216)	PTD (113)	CTD (74)	TS (29)	P
types of blinking	excessive and frequency blinking	95 (43.98%)	51 (23.61%)	27 (12.50%)	17 (7.87%)	χ^2^ = 8.568 P=0.380
	frequency blinking	83 (38.43%)	42 (18.58%)	26 (12.03%)	15 (6.94%)	
	excessive blinking	71 (32.87%)	34 (15.71%)	26 (12.03%)	11 (5.09%)	
	eye eye rolling	47 (21.76%)	18 (8.33%)	21 (9.72%)	8 (3.70%)	
	others	37 (17.13%)	23 (10.65%)	12 (5.55%)	2 (0.93%)	
allergic diseases	AR	120 (55.56%)	64 (29.63%)	35 (16.20%)	21 (9.72%)	χ^2^ = 22.456 p<0.001
	allergic asthma	84 (38.89%)	35 (24.14%)	12 (5.55%)	37 (17.13%)	
	others	20 (9.26%)	11 (4.66%)	7 (4.73%)	2 (3.77%)	
allergens	number of allergens tested	98 (45.37%)	41 (16.27%)	37 (25.0%)	20 (30.73%)	
	dust mites	37 (17.13%)	14 (6.48%)	15 (6.94%)	8 (3.70%)	χ^2^ = 0.762 P=0.943
	milk/beef	11 (5.09%)	5 (2.31%)	4 (1.85%)	2 (0.93%)	
	others	16 (7.41%)	8 (3.70%)	5 (2.31%)	3 (1.39%)	

**Table 8:** Clinical characteristics of DED in TD patients.

Characteristic		TD (n=145)	PTD (72)	CTD (54)	TS (19)	P
types of blinking	excessive and frequency blinking	47 (32.41%)	23 (15.86%)	18 (12.41%)	6 (4.14%)	χ^2^ = 5.414 P=0.713
	frequency blinking	51 (35.17%)	26 (17.93%)	17 (11.72%)	8 (5.52%)	
	excessive blinking	32 (22.07%)	17 (11.72%)	12 (8.28%)	3 (2.07%)	
	eye eye rolling	22 (15.17%)	7 (4.83%)	7 (4.83%)	4 (2.76%)	
	others	15 (10.34%)	10 (6.89%)	2 (1.38%)	3 (2.07%)	
symptoms and signs	both signs and symptoms	62 (42.76%)	36 (24.83%)	21 (14.48%)	5 (3.45%)	χ^2^ = 4.857 P=0.302
	Only symptoms	47 (32.41%)	33 (22.76%)	11 (7.59%)	3 (2.07%)	
	Only signs	36 (24.83%)	18 (12.41%)	15 (10.34%)	3 (2.07%)	

#### Neurological and psychiatric disorders

Overall, varying severities of neurological and psychiatric disorders were exhibited by 267 patients (58.94%), including 183 (40.40%) with ADHD, 72 (15.89%) with OCD, 147 (32.45%) dysthymic disorder (e.g., irritability, anxiety, fear, and/or depression), and 35 (7.73%) with social dysfunction. Analysis via Chi-squared tests revealed significant differences in neurological and psychiatric disorder prevalence according to TD subtype; TS patients had the highest incidences of all types of neurological and psychiatric disorders (P<0.01, P<0.05). Details are presented in **Table 9**.

**Table 9:** Neurological and psychiatric disorders.

Characteristic		TD (n=453)	PTD (252)	CTD (148)	TS (53)	P
neurologic and psychiatric disorders	ADHD	183 (40.40%)	90 (35.71%)	59 (39.86%)	34 (64.15%)	χ^2^ = 14.733 P=0.001
	OCD	72 (15.89%)	29 (11.51%)	24 (16.22%)	19 (35.85%)	χ^2^ = 19.426 P<0.001
	dysthymic disorder	147 (32.45%)	72 (28.57%)	54 (36.49%)	26 (49.06%)	χ^2^ = 9.090 P=0.011
	social dysfunction	35 (7.73%)	13 (5.16%)	10 (6.76%)	12 (22.64%)	χ^2^ = 19.064 P<0.001

### Therapy and follow-up

In total, 317 TD patients with eye diseases were treated according to the guidelines for their specific conditions. Of these patients, 256 (80.76%) experienced improvement or resolution of their tic symptoms. Among the 141 patients with suspected TD, 85 had eye diseases: 54 exhibited persistent eye or other tics after treatment and were ultimately diagnosed with TD, whereas 31 experienced symptom reduction after ocular disease treatment, leading to the exclusion of a TD diagnosis. Among the 56 patients who presented with abnormal blinking alone, 32 developed other tics or experienced persistently worsening abnormal blinking, resulting in a diagnosis of TD. Abnormal blinking spontaneously resolved in the remaining patients, leading to the exclusion of a TD diagnosis during the 1-year follow-up period. Additionally, 45 PTD patients displayed progression to CTD or TS during follow-up. In 236 TD patients with ametropia, tic symptoms did not improve after refractive correction.

## Discussion

The present study aimed to investigate the clinical characteristics of TD in children and adolescents presenting with the chief complaint of abnormal blinking. Our findings underscore the importance of recognizing TD as a significant cause of abnormal blinking in this population, especially considering the high prevalence of TD (42.98%) among our cohort. Meanwhile, our results showed that 18.98% of patients were diagnosed during the follow-up, and 26.71% of patients were initially misdiagnosed or experienced a missed diagnosis. This result emphasizes that ophthalmologists need to be vigilant about the possibility of TD when managing children with abnormal blinking, particularly when accompanied by tics other than eye. We also found that PTD patients were more likely to visit an ophthalmology clinic and patients with CTD or TS were more likely to visit a paediatrician. Similarly, we found that CTD and TS had more tic symptoms, more neurological and psychiatric disorders, and were more likely to have a history of abnormal blinking or TD. Taken together, these results indicate that the symptoms of CTD and TS are usually more severe than those of PTD. Parents of PTD patients with abnormal blinking often initially choose to consult a paediatric ophthalmologist, when those patients combined with eye diseases, misdiagnosis or missed diagnosis is more likely. Therefore, ophthalmologists should consider the possibility of TD when children present with blinking-related complaints, even in the presence of eye diseases. Referral to a specialist is warranted when patients exhibit repeated blinking or have a history of TD.

Among the TD patients, PTD was the common TD subtype (252, 55.63%), indicating that most patients had a short course of disease. The tic symptoms primarily involved eye and facial tics, and tic types were mainly motor tics and simple tics, including excessive blinking, frequent blinking, eye rolling, nose wrinkling, mouth tics, and facial grimacing; complex tics were less common, only 22 children displayed coprolalia and four exhibited aggressive behaviour, which also explains why TD patients often seek ophthalmologic care and are misdiagnosed with an eye disease. This finding aligns with previous studies reporting a higher incidence of PTD compared to CTD and TS (5). However, many children also exhibited nose and throat clearing, which may be related to the tendency of TD patients visiting the ophthalmology clinics to have comorbid AC or AR. Besides, neurological and psychiatric disorders were not uncommon. In our cohort, 40.40% of TD patients had ADHD, whereas 32.45% had dysthymic disorder. TS patients had the highest incidences of neurological and psychiatric disorders. These findings are consistent with previous studies, indicating a strong link between TD and these conditions (19, 20). The higher incidence of these disorders in TD patients highlights the need for a thorough neuropsychiatric evaluation in all TD patients, even those with PTD who visit ophthalmology clinics.

In our cohort, a notable finding was the high prevalence of comorbid eye diseases among TD patients, accounting for 74.17%, with AC and DED being the most common. Moreover, 81.90% of TD patients experienced ocular discomfort, the main symptoms were eye itching, dryness, redness, and fatigue, more patients had symptoms but were not diagnosed with eye diseases, which was related to prodromal tics. Tic symptoms showed varying degrees of improvement when DED and/or AC were treated. Therefore, a diagnosis of TD should not be excluded in patients presenting with the chief complaint of blinking and ocular discomfort, even if an ocular disease is diagnosed. Notably, 236 patients with ametropia did not experience any improvement in tic symptoms after refractive correction, suggesting that TD are more strongly associated with DED and AC than with ametropia.

There is increasing evidence that TD are closely associated with allergic diseases, TD patients with allergic diseases have higher motor and vocal tic scores than such patients without allergic diseases (21). Yuce et al. (22) discovered strong correlations between AR or allergic eczema and TS, the presence of allergic diseases is associated with tic symptoms and TD severity (23–25). Chen et al. (26) also found that patients with TD had significantly higher prevalences of allergic diseases (e.g., AR, atopic dermatitis, and AC). Considering that children with TD often exhibit conditions such as AR, allergic eczema, or AC, and the clinical courses of TD and allergic diseases are similar, exhibiting cycles of exacerbation and remission (25), TD with allergic diseases may be a primary cause of abnormal blinking. Our results suggest that the number of patients with TD combined with allergic diseases is much higher than that of patients with TD alone, and TD patients combined with AC exhibit more severe tic symptoms. Additionally, in clinical practice, we have observed that effective control of allergic diseases can facilitate the prevention and management of TD, which is consistent with previous studies (23–25).

The aetiologies of allergic diseases are complex, involving a combination of genetic factors, allergens, and various physical and chemical factors, they mirror the complexity of TD aetiologies (27). Allergic diseases can cause considerable psychological stress for patients, which has emerged as a strong predictor of the severities of tics, OCD, and depressive symptoms. Moreover, stress can exacerbate the symptoms of both TS and allergic diseases (28). Additionally, TD and various allergic diseases exhibit abnormalities in inflammatory markers such as interleukins and tumour necrosis factor, suggesting shared immune responses. Allergy-associated immune dysfunction may increase the severities of tics and other abnormal behaviours in children with TD (29). The results of some immunological and genetic studies have supported this link. For example, Hartmann et al. (30) reported elevated histamine levels in patients with both TS and allergic diseases. Chen et al. (23) not only found the close relationship between PTD and AC, but also suggested that PTD is an entity distinct from other TD subtypes, such as TS, but a potential link between allergies and tic symptoms, warranting further investigation. All of these findings support a connection between TD and allergic diseases.

There is also evidence of a strong correlation between abnormal blinking and DED (31) and higher prevalence rates of DED in children have been reported (32). Even minor abnormalities in the ocular surface and tear film can increase blinking rates. Frequent blinking is common in children with AC, especially when accompanied by DED (33). In our clinical practice, we have identified both TD and DED in some patients who present with abnormal blinking as the chief complaint. Chen et al. (23) reported that AC was less prevalent than DED in PTD patients. Those results indicated that the incomplete blinking induced by eye tics in children with TD led to tear film instability and DED. In children with TD, eye tics often involve frequent and excessive blinking. This can result in inadequate tear lipid distribution and subsequent exposure of the inferior ocular surface, potentially increasing tear evaporation (34). Moreover, incomplete blinking has been associated with decreased TFBUT, increased ocular surface disease index scores, and insufficient lipid layer thickness (35). Patients with incomplete blinking reportedly have more severe meibomian gland loss and reduced tear film stability (36). Therefore, in TD comorbid with DED patients, on the one hand, incomplete blinking result in instability of the tear film; On the other hand, a decrease in meibomian gland secretion capacity leads to the patients squeezing the eyes hard and increasing blinking rates, those abnormal blinking habits can aggravate TD.

AC and DED, common triggers of TD in children, can occur alone or in combination in TD patients. The recurrent nature of AC in children with TD can lead to tear film instability and ocular inflammation, predisposing them to DED. Chen et al. (32) reported that the frequency of DED, as determined by TFBUT, was 97.5% in young children with AC. Additionally, an AC-associated DED was solely characterised by decreased TFBUT, affected individuals remained asymptomatic (37, 38). In the present study, tear film instability was evident in 24.07% of TD with AC patients, but more than half of these children did not report any dry eye-related symptoms.

As we have discussed above, abnormal blinking is one of the most common symptoms shared by AC, DED, and TD, which can hinder effective differential diagnosis. This comorbidity underscores the complexity of managing patients with abnormal blinking and Children with TD are often misdiagnosed with conditions such as AC, DED. On the basis of our extensive clinical experience, we believe that the first step towards accurate diagnosis involves gathering information about the clinical manifestations, including the types of abnormal blinking, blink rate and duration, presence of accompanying facial or other tics, and any past medical history of blinking or tics. Generally, excessive blinking accompanied by facial and other tics, and/or recurrent abnormal blinking are features that suggest the presence of TD. The second step involves identifying the cause of abnormal blinking. A detailed history should be collected, an ophthalmic examination should be performed to determine whether the patient exhibits ocular symptoms and signs of eye diseases. If no ocular discomfort is present, the abnormal blinking is likely caused by TD. If ocular discomfort is obvious, further evaluation is needed to determine whether the abnormal blinking is caused by eye diseases or a combination of eye diseases and TD. Third, in patients with both TD and eye diseases such as AC, abnormal blinking usually improves but does not completely resolve after appropriate treatment of the ocular diseases. Namely, children with abnormal blinking should be evaluated for TD when blinking persists despite adequate treatment for eye diseases. Finally, when AC is accompanied by AR and asthma, excessive blinking, nose wrinkling, throat clearing, and other symptoms can be difficult to distinguish from TS, and many children are missed diagnosed, adequate treatment of AC and/or AR can help identify TD. It also can prevent the exacerbation of tic symptoms. Therefore, a detailed medical history should be collected and a physical examination (including mental assessments and necessary auxiliary examinations) should be performed along with diagnostic treatment and follow-up as needed to ensure accurate diagnosis of TD, particularly in patients comorbid with allergic diseases. Our study established diagnostic principles to differentiate between ocular disease-related and TD-associated abnormal blinking, emphasizing the importance of follow-up on tic symptoms and eye disease management, and considering the persistence or occurrence of other tic symptoms.

It is worth noting that the symptoms of some eye diseases are easy to be confused with the prodromal symptoms of TD (i. e., sensory tics), such as itching and pain. Therefore, we need to determine whether these symptoms are caused by eye disease or are prodromal symptoms of TD before diagnosing eye diseases. The consistency of ocular symptoms and signs is key to differentiation. When a patient's ocular discomfort is consistent with the signs, we tend to diagnose eye diseases. However, when a patient's ocular discomfort is inconsistent with the signs or only ocular discomfort, we consider the prodromal symptoms of TD and follow-up. Despite strict differentiation, a very small number of patients may still be misdiagnosed due to the complex connections among these diseases.

To our knowledge, this is the largest cohort of TD patients diagnosed in an ophthalmology setting to date (7–10). Our results provide an accurate summary of the clinical characteristics of children with TD who present with the chief complaint of abnormal blinking. Thereas this study had some limitations. First, we did not perform allergen tests in all patients with suspected AC; instead, we relied on their allergic disease histories and typical ocular signs. Second, because TD can spontaneously resolve in patients with AC or DED, even if all symptoms disappeared during treatment and follow-up, TD could not be definitively excluded. Therefore, we may have underestimated the prevalence of TD in patients with abnormal blinking. Third, allergic diseases and DED are influenced by climate and geographic factors, the inclusion of patients only from southeast China may limit the generalisability of our findings to other populations. Finally, our follow-up period is limited, Longitudinal studies with longer follow-up periods are needed to better understand the natural history of TD.

## Conclusions

This is the largest cohort study of TD patients in an ophthalmology setting to date, our study highlights the significance of TD as a cause of abnormal blinking in children and adolescents, and PTD is the most common subtype. The high prevalence of comorbidities between TD and eye diseases, mainly AC and DED, increases the complexity of managing these patients, who are likely to be misdiagnosed or experience missed diagnosis. Finally, this study highlights the fact that abnormal blinking is a common symptom shared by TD, AC, and DED, our study established diagnostic principles to differentiate between ocular disease-related and TD-associated abnormal blinking, emphasizing the importance of follow-up on tic symptoms and eye disease management.

## Data Availability

The raw data supporting the conclusions of this article will be made available by the authors, without undue reservation.
